# Multispectral Wavebands Selection for the Detection of Potential Foreign Materials in Fresh-Cut Vegetables

**DOI:** 10.3390/s22051775

**Published:** 2022-02-24

**Authors:** Salma Sultana Tunny, Hanim Z. Amanah, Mohammad Akbar Faqeerzada, Collins Wakholi, Moon S. Kim, Insuck Baek, Byoung-Kwan Cho

**Affiliations:** 1 Department of Biosystems Machinery Engineering, College of Agricultural and Life Science, Chungnam National University, 99 Daehak-ro, Yuseong-gu, Daejeon 34134, Korea; sstunny@o.cnu.ac.kr (S.S.T.); hanim_za@ugm.ac.id (H.Z.A.); akbar.faqeerzada@gmail.com (M.A.F.); wcoln@yahoo.com (C.W.); 2 Department of Agricultural and Biosystems Engineering, Faculty of Agricultural Technology, Gadjah Mada University, Yogyakarta 55281, Indonesia; 3 Environmental Microbial and Food Safety Laboratory, Agricultural Research Service, United States Department of Agriculture, Powder Mill Road, BARC-East, Bldg 303, Beltsville, MD 20705, USA; moon.kim@usda.gov (M.S.K.); insuck.baek@usda.gov (I.B.); 4 Department of Smart Agriculture Systems, College of Agricultural and Life Science, Chungnam National University, 99 Daehak-ro, Yuseong-gu, Daejeon 34134, Korea

**Keywords:** fresh-cut vegetables, foreign materials, near infrared spectroscopy, waveband selection

## Abstract

Ensuring the quality of fresh-cut vegetables is the greatest challenge for the food industry and is equally as important to consumers (and their health). Several investigations have proven the necessity of advanced technology for detecting foreign materials (FMs) in fresh-cut vegetables. In this study, the possibility of using near infrared spectral analysis as a potential technique was investigated to identify various types of FMs in seven common fresh-cut vegetables by selecting important wavebands. Various waveband selection methods, such as the weighted regression coefficient (WRC), variable importance in projection (VIP), sequential feature selection (SFS), successive projection algorithm (SPA), and interval PLS (iPLS), were used to investigate the optimal multispectral wavebands to classify the FMs and vegetables. The application of selected wavebands was further tested using NIR imaging, and the results showed good potentiality by identifying 99 out of 107 FMs. The results indicate the high applicability of the multispectral NIR imaging technique to detect FMs in fresh-cut vegetables for industrial application.

## 1. Introduction

Fresh-cut fruits and vegetables refers to ready-to-eat or ready-to-cook plant products that have been slightly physically modified by slicing, cutting, peeling, or trimming and then packed as 100% edible products. The International Fresh-Cut Produce Association (IFPA) reported that since 1930, fresh-cut products have been available in retail supermarkets [1]. In the beginning, fresh-cut production companies were established for supplying products to hotels, restaurants, and catering services. However, this industry has become popular in the last few years because of the growing need for fresh fruit and vegetables for consumption [2].

Now, fresh-cut fruit and vegetables are among the most popular commodities in industrialized countries’ food markets, remaining the most demanded plant-derived products to meet the needs of modern consumers. Moreover, in the last decade, because of technological advancements, this specialized industry has been successively growing and is better organized [3].

The main challenge of this industry is to ensure the freshness and quality of the fresh-cut products. Quality is dependent upon physical, chemical, and biological contamination. Several studies have been conducted regarding chemical and biological hazards and changes in fresh-cut produce [4,5,6,7]. Fecal contamination and different types of foreign materials (FMs) are the major concerns of physical contamination [7]. In physical contamination, a wide range of foreign materials has been the main source of consumer complaints in the fresh-cut industry. A comprehensive study conducted as a part of the Food Standards Agency (UK) showed that around 20% of incidents concerning foreign materials were reported for vegetables and vegetable products, which is the highest among all food products. The study also found that plastics, glass, metal, wood and plant parts, and insects are the most frequent foreign materials found in vegetables [8].

The presence of FMs in food can cause health risks for the consumer and also affect the reputation of the manufacturer. It is mandatory to ensure the removal of all types of foreign bodies from foods. Several studies have been conducted to identify FMs in different types of food, including X-ray, near infrared spectroscopy, hyperspectral imaging, thermal imaging, ultrasonic, and terahertz methods [9]. Metal detectors, magnets, electrical impedance, and surface-penetrating radar are some of the commonly used methods of the different food processing industries. However, these methods are only suitable for specific types of FMs.

Though FMs are mostly found in vegetables and vegetable products, no study has been performed to evaluate the performance of the systems mentioned earlier for fresh-cut vegetables. Being concerned about the seriousness of foreign materials incidents in fresh-cut vegetables, fundamental research is needed to elucidate a suitable method to identify FMs in fresh-cut produce. A previous study found that the X-ray system is not ideal for detecting plastic fragments in food, and it is also costly [10]. Ultrasonic imaging is not appropriate in the air medium [11], and terahertz imaging is greatly attenuated in the water medium [12]. To use a thermal imaging system, a thermal contrast between the product and the background is needed [13], and no temperature interference from other surfaces is acceptable [11].

In contrast, NIR can penetrate the air spaces between food materials [12], and it is also cheaper, easier, and faster than the other techniques [14,15]. The main disadvantage of NIR spectra is that they often contain overlapping peaks caused by the overtone and combination of the fundamental vibration of the molecules, which draws on broad bands [16]. This makes the spectra challenging to interpret, and extracting chemical information is less straightforward. However, this problem can be avoided by using proper chemometric techniques to build a relationship between absorption values at certain wavelengths and reference values of the measured samples [17]. Another limitation of the NIR technique when using chemometrics is that the technique requires reference data during calibration [17]. However, to classify between two categorical groups, it is easy to use “0” and “1” as the reference values for calibration purposes, which can easily overcome the limitations of NIR.

Two different types of chemometric approaches are used for the classification of NIR spectra, namely the unsupervised and supervised methods [18]. PCA is one of the most common unsupervised techniques in which similar materials have a tendency to create a cluster in the multidimensional plot based on their principal component scores [19]. On the other hand, PLS-DA is a popular supervised chemometric method for analyzing high-dimensional data, where the variables are often correlated with one other [20]. In addition, the algorithm is computationally inexpensive, which is preferable for industrial use [21].

Therefore, an NIR imaging system with proper chemometric methods could be a solution for identifying FMs in fresh-cut vegetables. Nevertheless, before designing the spectral imaging system, it is essential to separately observe the spectral characteristics of potential FMs and vegetables and select critical wavebands to differentiate them. Furthermore, new applications appear in the food industry on a daily basis due to the introduction of noninvasive, chemical free, and fast-moving technologies [22]. Previously, the NIR imaging system was used to identify leaves, twigs, and stones in blueberries [23]; coins, glass balls, and rubber in dough, cheese, doughnuts, and meats [12]; leaves and stems in blueberries [24]; bone fragments in chicken breast fillets [25]; and polyethylene terephthalate (PET), polyethylene (PE), metal, insects, and bones in pork steaks [26]. Additionally, near infrared spectroscopy was used to identify insects in various foods, such as chestnuts [27], rice [28], and olives [29]. However, each study considered very few selected materials as FMs.

This paper assesses the feasibility of detecting a wide range of FMs in fresh-cut vegetables based on NIR spectroscopy with multivariate analysis. The primary objective of this research is to establish the crucial wavebands that can separate the potential FMs from different types of fresh-cut vegetables and explore the limitations of NIR application in detecting FMs.

## 2. Materials and Methods

### 2.1. Samples

**Figure 1:**
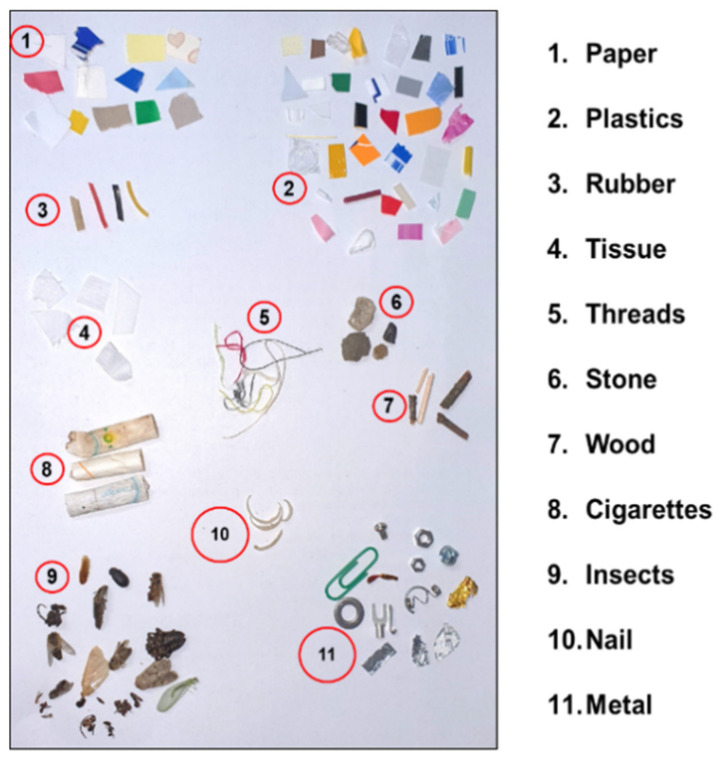
Photograph of representative FMs.

Seven types of vegetables, namely cabbage, carrot, radish, green onion, onion, potato, and zucchini, were used in this study. All of the vegetables were purchased fresh from the local market of Daejeon in the Republic of Korea, and then cut into small pieces, with the spectra measured on the same day. For each type of vegetable, 40 sample spectra were collected, i.e., a total of 280 sample spectra. Different types of plastics, wood, stone, bugs, paper, tissue, nails, rubber, etc., were considered as FMs. Figure 1 shows the representative FMs, and a list of potential FMs is provided in Table 1. A total of 320 spectra were collected from all types of FMs.

### 2.2. NIR Spectroscopy

The spectra of all the vegetables and FMs were acquired using an FT-NIR spectrometer (Antaris II FT-NIR analyzer, Thermo Scientific CO., Waltham, MA, USA), which was set with an InGaAs detector. The sampling window consisted of chemically resistant sapphire. Each piece of vegetable or FM was put in the middle of a custom-made sample holder with a scanning glass (low OH quartz) window designated for solid samples, which helped to ensure the reproducibility of the diffuse reflectance spectra. The holder had a black lid to restrict the outside light effect. The absorbance spectra of each piece were collected using a total of 32 successive scans in the wave range of 1000–2500 nm (4000–10,000 cm^−1^) at an interval of 4 cm^−1^ spectral resolution. Large pieces of FMs and vegetables were cut into small pieces to fit the sampling area properly. Then, the spectra of each piece of vegetables and FMs were obtained separately.

### 2.3. NIR Imaging System

A laboratory-based line scan NIR imaging system was used in this study to acquire the imaging data in the wave range of 900–2500 nm. The system consisted of an imaging spectrograph (SWIR, Headwall Photonics, Fitchburg, MA, USA), an MCT (mercury cadmium telluride) detector (Xeva-2.5-320, Xenics, Belgium) with a camera of 320 × 256 pixel resolution, a 25 mm f/1.4 (OB-SWIR25/1.4—P/N C0808.010) lens, and six 100 W tungsten-halogen light sources. The sample was moved at 4.73 mm/scan on the way to the camera field of view by a translation stage controlled by a DC motor.

### 2.4. Spectral Data Preprocessing and Multivariate Analysis

Because of the scattering of the light, changes in particle size, and instrumental drift, the FT-NIR spectra may have had noise. Therefore, the raw data were required to undergo preprocessing methods to attain precise chemical information from the spectral peaks and valleys [30]. In this study, all spectral data were treated with seven preprocessing methods: three data normalization methods (maximum, minimum, and range), two derivatives (Savitzky–Golay first and second), multiplicative scatter correction (MSC), and standard normal variate (SNV).

To understand the characteristics of the spectral data and the extent of data diffusion, principal component analysis (PCA) could be easily applied. The PC1 (first principal component) explains the most significant variation in the data. Then, a PC2 (second principal component), which is orthogonal to the first PC, defines the reamining variation, which PC1 does not describe, and so on. The most significant characteristics of the spectral data can be observed on a graphical plot using this tool [31].

Afterward, a multivariate analytical model of PLS-DA was developed to discriminate the vegetables from the different types of FMs. PLS-DA, typically applied for model classification, is the modified form of partial least square regression (PLS-R). It is expressed as:

(1) Y=X×β+ė

where X is an m×n matrix that contains the predictor variables of each class, β is the vector of the regression coefficient, ė is the error term, and Y is the response vector comprised of a synthetic value expressing class, as provided below:

$$Y=\left\{\begin{array}{c} 0=\mathrm{sample}\;\mathrm{aligned}\;\mathrm{to}\;\mathrm{vegetable}\;\mathrm{class}\;\\ 1=\mathrm{sample}\;\mathrm{aligned}\;\mathrm{to}\;\mathrm{foreign}\;\mathrm{materials}\;\mathrm{class} \end{array}\right.$$

In order to precisely distinguish the vegetables and FMs, a baseline was chosen as ± 0.5 with respect to each class. Then, the X and Y values were decomposed into latent variables (LVs) to establish a linear connection between the response and predictor variables.

(2) $$X={\mathrm{TP}}^{T}+E_{x}$$

(3) $$Y={\mathrm{UQ}}^{T}+E_{y}$$

where T and U are score matrices, and whereas P and Q are loading matrices. E_x_ and E_y_ are the error matrices of X and Y, respectively.

### 2.5. Overview of Multispectral Waveband Selection Methods

The main objective of an important variable selection is to extract the most valuable wavebands from all of the spectral data, which can discriminate the FMs from vegetables, while removing the redundant bands. This will make the model more suitable for industrial applications by significantly reducing the computational time. In this study, five optimal variable selection methods were used, namely weighted regression coefficient (WRC), variable importance in projection (VIP), sequential feature selection (SFS), successive projection algorithm (SPA), and interval PLS (iPLS).

#### 2.5.1. Waveband Selection Based on WRC

The weighted regression coefficient (weighted β-coefficient) shows the connections between the predictors and the corresponding response variables by measuring their weight. The WRC was obtained from the PLS-DA model by dividing each spectrum by its standard deviation to adjust the data on the same scale. The higher positive or negative values of the β-coefficient represent the essential variables that contribute the most to build the model. It is a widespread method for selecting wavelengths and used successfully by many researchers [32,33,34]. However, the absorbance value from the original sample spectra needs to consider the corresponding WRC value to select the variables. Variables with a higher β-coefficient but lower (or no) peak in the sample spectra may not contribute to the model prediction [35].

#### 2.5.2. Waveband Selection Based on VIP

Variable importance in projection, as described in [36], was also applied as an optimal variable selection method to build a classification model to differentiate between vegetables and FMs. The general equation to calculate the VIP score of variable j is given below:

(4) $${\mathrm{VIP}}_{j}=\sqrt{\frac{\sum_{i=1}^{I}W_{\mathrm{ji}}^{2}{\;\mathrm{SSY}}_{i}\;J}{{\mathrm{SSY}}_{\mathrm{total}}\;I}}\;$$

where $W_{\mathrm{ji}}$ is the weight value for the element i and the variable j, ${\mathrm{SSY}}_{i}$ is the sum of the squares of the measured variable for the ith element, J is the total number of variables, ${\mathrm{SSY}}_{\mathrm{total}}$ is the overall sum of squares measured for the response variable, and I is the total quantity of the elements.

#### 2.5.3. Waveband Selection Based on SFS

Sequential feature selection is a method that selects features one-by-one in an iterative way by checking the best model fit. It mainly has two components: the criterion function and a sequential search algorithm. The algorithm starts with an empty set and then sequentially adds features, one-by-one, that significantly describe the model based on the criterion function [37]. The criterion function can be defined in two ways: for the classification model, the criterion function is a misclassification rate, and for the regression model, it is the mean squared error. The searching algorithm continues the same process until the required number of variables is selected.

#### 2.5.4. Waveband Selection Based on SPA

The successive projection algorithm was used to select the optimum number of variables by minimizing the redundancy information produced by the spectroscopy to improve the collinearity problems among the variables [38]. The algorithm starts with a single wavelength or variable, then adds a new variable with the maximum projection, and repeats the process until the desired number of variables is selected. Thus, the algorithm produces a subset of variables with the least linear relationship between them from all the variables of the training set and applies this set to the cross-validation data set to evaluate the performance. This algorithm is extensively used to select the optimum number of variables in the multivariate quantitative and qualitative analysis [34].

#### 2.5.5. Waveband Selection Based on iPLS-DA

iPLS-DA is a modified version of the iPLS algorithm described in [39], which identifies the critical variables from the entire spectrum region by dividing them into equal subintervals and then finding the minimum misclassification rate. The model can be developed in two selection modes: forward and backward. In the forward selection mode, the intervals are added in an iterative way until the user-defined conditions are fulfilled. On the other hand, the intervals are removed iteratively in the backward selection mode. However, the backward selection mode typically selects more variables than the forward one [40]; therefore, the forward selection model was used in this study.

All preprocessing methods, variable selection algorithms, and PLS-DA model present in this study were executed using MATLAB R2020b (MathWorks, Natick, MA, USA) computer software.

### 2.6. Model Performance Assessment

Sensitivity, specificity, and accuracy were calculated to assess the performance of the model developed with the selected variables by using the following equations adapted from Faqeerzada et al., 2020 [41].

(5) $$\mathrm{Sensitivity}\left(\%\right)=\frac{\mathrm{Tp}}{\mathrm{Tp}+\mathrm{Fn}}\times 100\;$$

(6) $$\mathrm{Specificity}\left(\%\right)=\frac{\mathrm{Tn}}{\mathrm{Tn}+\mathrm{Fp}}\times 100\;$$

(7) $$\mathrm{Accuracy}\left(\%\right)=\frac{\mathrm{Tp}+\mathrm{Tn}}{\mathrm{Tp}+\mathrm{Tn}+\mathrm{Fp}+\mathrm{Fn}}\times 100\;$$

where Tp = True positive (sum of accurately detected vegetable samples), Fn = False negative (sum of the vegetable samples detected as FMs), Tn = True negative (sum of the accurately categorized FMs), and Fp = False positive (sum of the FMs grouped as vegetables).

## 3. Results and Discussion

### 3.1. Spectral Data Interpretation

**Figure 2:**
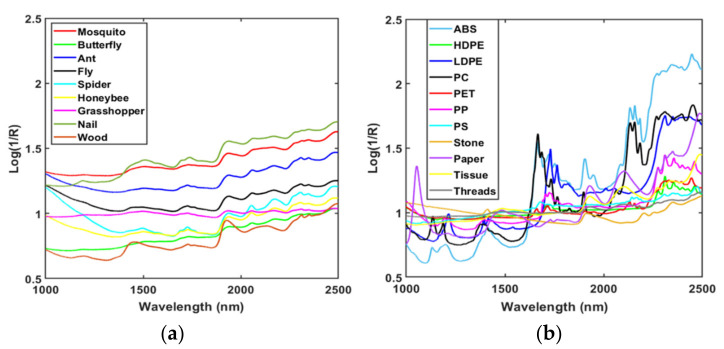

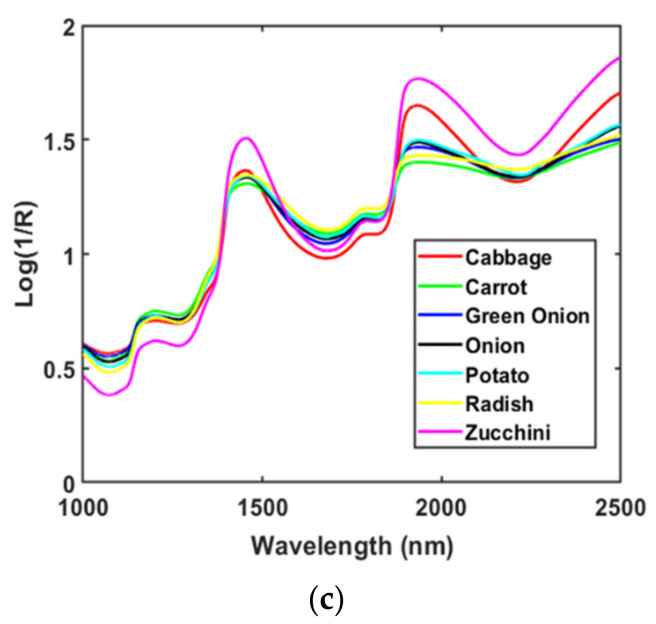
Raw FT-NIR spectral plots of (**a**) biological FMs, (**b**) non-biological FMs, and (**c**) fresh-cut vegetables.

The averages of the raw spectral data of the fresh-cut vegetables and all FMs were plotted for the full NIR wavelength (1000–2500 nm) region. In order to make these spectral features easy to comprehend, spectra were plotted in three sections: biological FMs spectra are displayed in Figure 2a, non-biological FMs spectra are shown in Figure 2b, and fresh-cut vegetable spectra are shown in Figure 2c. The spectra in the wavelength regions of 1400–1450 nm and 1900–1950 nm show higher intensity for vegetables than the FMs due to the presence of excessive water content, which can differentiate the fresh-cut produce from foreign bodies. The absorption bands of vegetables found at 1190 nm, 1450 nm, and 1940 nm were assigned to the combinations of the O–H first overtone and O–H bending band, the first overtone of O–H stretching band, and the combination of O–H stretching band and O–H bending band, individually [42].

However, it was observed that almost all of the FMs and vegetables had a peak near 1700–1800 nm, with different intensities. Several studies have found that the absorption band detected close to 1700–1800 nm due to the first overtone of C–H stretch proves the presence of cellulose content in vegetables, bugs, papers, and woods [43,44,45]. In addition to this, plastics (LDPE, HDPE, PP, ABS, and PET) also have peaks over 1720–1770 nm due to the first overtone of the asymmetric stretching of a methyl group [46]. As the main purpose of this study was to classify the FMs in fresh-cut vegetables, the bands with no similarity or conflict with vegetable spectra were not discussed.

### 3.2. Principal Component Analysis (PCA)

Spectral datasets typically contain many more variables than the number of samples, which can make a model overly confident when multiple linear regression techniques are used [47]. As an unsupervised approach, the principal components in a PCA model can serve as an indicator for model reliability by pointing out the direction of maximum variance [48]. The score plot of the PCA model is a subspace projection of data that is used to analyze the relationships between the observations.

**Figure 3:**
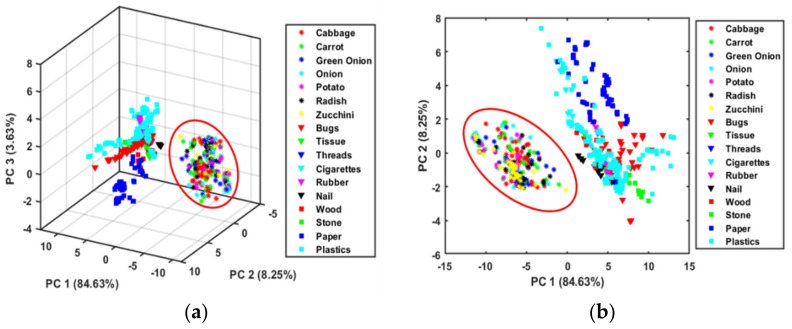

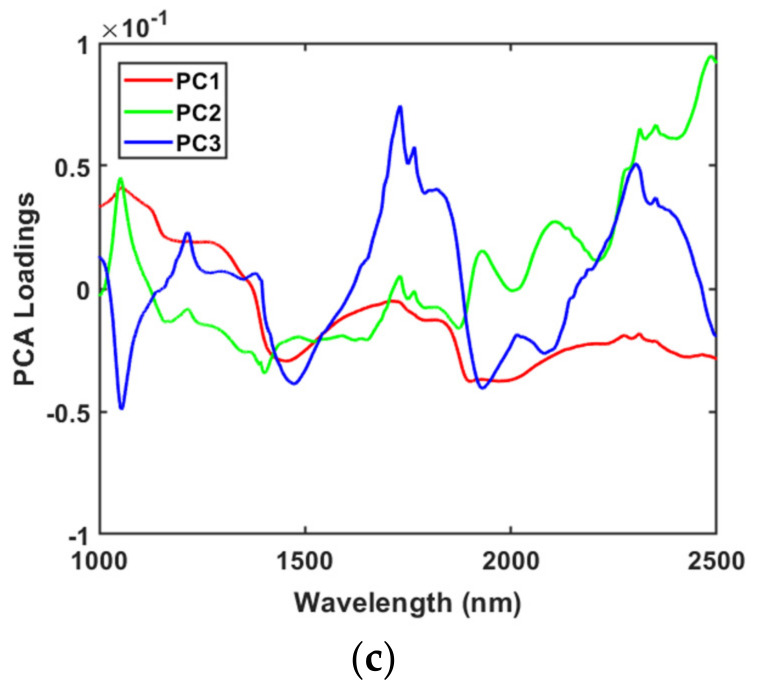
(**a**) First three PC score plot, (**b**) first two PC score plot, and (**c**) PCA loadings plot of mean normalized spectra.

The obtained scatter plot of the PC scores after applying the mean normalization preprocessing method, displayed in Figure 3a,b, shows significant variation between the vegetables and FMs. The first three PCs demonstrated a cumulative total variation of 96.51% in the classification of FMs and vegetables. As demonstrated in the two-dimensional projection (Figure 3b), PC1 and PC2 were the most important components for FM identification, contributing a total variation of 92.9%.

The PC loading plot was developed to define the corresponding weights of the variables for the first three PCs (PC1, PC2, and PC3), as shown in Figure 3c. These loading plots showed some critical information about wavelengths and had similarities with the peaks described in the previous section. Three peaks positioned at 1450 nm, 1730 nm, and 1920 nm may have a maximum contribution to discriminate the FMs from vegetables. The disparity between vegetables and FMs using PCA and the existence of spectral peaks at the specified wavelength established the base for the following study to construct the classification model using PLS-DA.

### 3.3. PLS-DA Model

Partial least square discriminant analysis (PLS-DA) was accomplished as a supervised learning model where two known classes were defined. The total data set was randomly shuffled and divided into two data sets where 70% of the data was used in the calibration model and the remaining 30% of data was used in model validation. The classification parameters to identify FMs in fresh-cut vegetables were acquired from the PLS-DA model after employing the preprocessing methods. A baseline shift and non-linear effects can be effectively avoided by using appropriate preprocessing to enhance the classification models [49]. Hence, the effect of different preprocessing methods on the accuracy of classification models was investigated and is summarized in Table 2.

**Figure 4:**
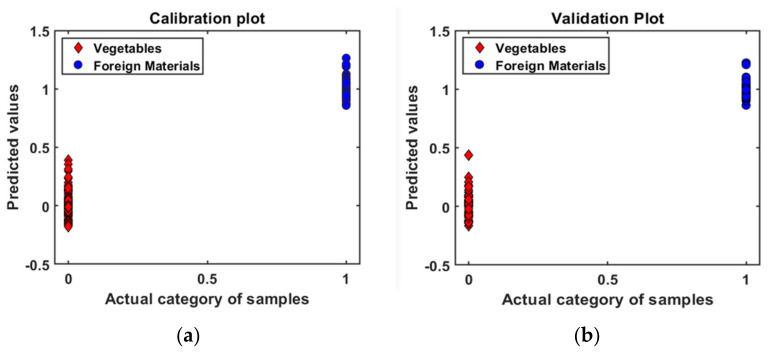
PLS-DA classification plots of raw data: (**a**) calibration plot and (**b**) validation plot.

The yielded PLS-DA models demonstrated maximum accuracy (100%) in calibration data sets, including all the preprocessing methods; in validation data sets, only the Savitzky–Golay second derivative (99.4%) had slightly lower classification accuracy. The raw data, i.e., data without any preprocessing, clearly classified the vegetables and FMs with a reference baseline of 0.5, both in the calibration and validation data, respectively (Figure 4). Sugiyama [24] was able to detect the leaves and stems from blueberries with 100% accuracy using NIR spectra with discriminant analysis. Diaz [26] clearly distinguished the PET, PE, metal, insects, and bone from pork steaks in the 1100–2500 nm wavelength region. The high classification accuracy indicated that there are apparent differences in chemical composition between the two groups, and FT-NIR spectroscopy could correctly and reliably identify the FMs in fresh-cut vegetables.

### 3.4. Selection of Important Wavebands

For the real-time application of a model, it is crucial to reduce the number of variables to run the model faster with better performance and reduce the associated cost. In addition, it is also helpful to develop a multispectral, low-cost imaging system that can ease the data collection and processing in a short time. Therefore, five extensively used variable selection algorithms, namely WRC, VIP, SFS, SPA, and iPLS, were used to select the most important variables.

**Figure 5:**
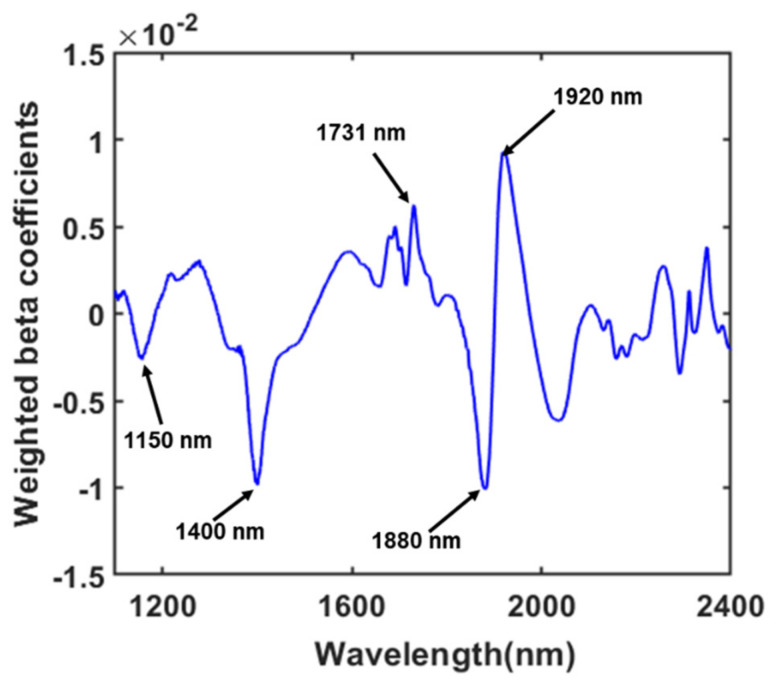
The beta coefficient curve obtained from the PLS-DA model.

Firstly, the weighted regression coefficient obtained from the PLS-DA model (Figure 5) was used to explore the connection strength between predictor and criterion variables. The peaks and valleys of the WRC represented the most valuable bands that contributed to classifying the FMs and vegetables. From Figure 5, five peaks and valleys with large weight values corresponding to specific wavelengths were selected, and the bands were 1150 nm, 1400 nm, 1731 m, 1880 nm, and 1920 nm.

The peaks around 1156 nm reflect the second overtone of the CH_3_ asymmetrical stretch associated with different plastics [46,50]. The wavelength at 1731 nm corresponding to the first overtone of CH stretching of cellulose is the main component of woods, papers, and bugs [43,44]. The absorption bands of foods containing 70–90% water are almost identical to those of pure water. Therefore, for the quantitative measurement of water content in foods, substantial FT-NIR absorption band ranges of approximately 1400–1450 nm and 1900–1950 nm were commonly used [51]. In this study, three strong peaks were found in the regression plot; among them, 1400 nm and 1920 nm corresponded to the moisture content of vegetables.

Secondly, the VIP, one of the well-known methods in multivariate analysis, was used to select the optimum wavebands. The number of predictors and the accuracy of the PLS-VIP model is usually determined by the cut-off value. The most excellent combination of model accuracy and number of variables was found at 1.4, which was chosen by exploring a range of values ranging from 0.8 to 1.5. After applying VIP analysis, the number of predictors drastically reduced from 1557 to 10, which was only 0.64% of the original FT-NIR spectral data. The selected wavelengths were in the 1881–1901 nm region, corresponding to the O-H structure.

Thirdly, the sequential feature selection, a wrapper method, was used to evaluate the selected features each time by calculating the error rate. This study calculated the error rate using a randomly selected 20-fold cross-validation method. The first selected wavelength was 1920 nm, and the error rate was 0.02%. After adding the second feature (1135 nm) to the dataset, the error rate became 0.004%, and finally, the misclassification rate became 0% when the third feature (1450 nm) was added to the dataset.

Fourthly, to select the important variables by SPA, the minimum and the maximum number of variables was selected (2 and 10, respectively), as the target was to select as minimal a wavelength as possible. Finally, the algorithm selected only four wavelengths (1300, 1402, 1925, and 2114 nm) without misclassification. The wavelength 1300 nm and 2114 nm are related to the CH bond in the lipid and carbohydrate, respectively [52,53].

Lastly, the forward selection interval PLS (iPLS) algorithm selected three wavelengths (1094, 1343, 1958 nm) using three intervals, with an interval width size of 1. The maximum number of the latent variable was five, and the model used cross-validation to evaluate the selected variables. The absorption band near 1094, 1343, and 1958 nm in the near-infrared region corresponds to the combination of CH, the first overtone, and the combination of the OH bond, respectively [34,52,54].

**Figure 6:**
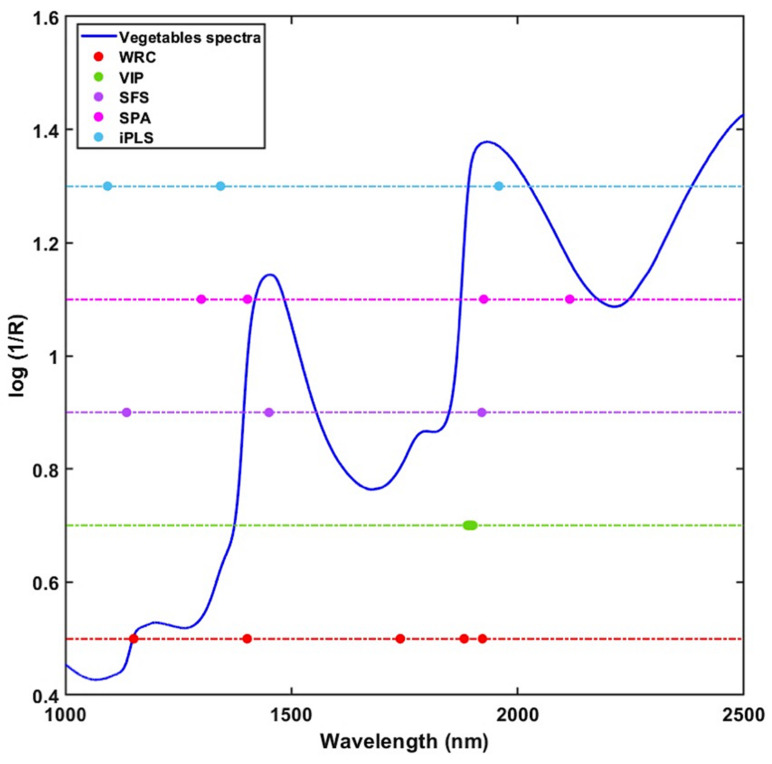
Graphical representation of the selected wavebands using the five variable selection methods.

However, each variable selection algorithm works based on different principles. Hence, the different variable selection approaches have shown considerable variation among the selected variables. Figure 6 and Table 3 show the wavelengths selected by each variable selection method. It can be seen that all the variable selection methods selected at least one variable in the range of 1900–1925 nm, except iPLS. In addition to that, WRC, SFS, and SPA selected a single wavelength in the 1400–1450 nm region. Several studies have proved that these two wavelength regions resemble the combination of the O-H bands associated with the water [35,51] present in the vegetables. However, both vegetables and FMs had some common peaks near the rest of the selected wavebands, but they had a clear difference in intensity. Therefore, it can be concluded that the models developed using these selected wavelengths are more robust and effective for the identification of FMs.

### 3.5. Model Performance Predictions Using Selected Wavebands

In order to prove the application of the selected wavebands, a new PLS-DA model was developed for each optimal variable selection method using raw spectra (without any preprocessing). Table 4 shows the performance of the new PLS-DA models developed using the selected variables. The results indicate that the new models (except VIP-PLS-DA) were able to differentiate the vegetables from the FMs with 100% accuracy (as with the original PLS-DA model (using all variables)). However, the VIP-PLS-DA model detected 93% of the FMs, while having the maximum number of variables (10) among the five variable selection methods. This could be because all the variables selected by the VIP method were in the range of 1888–1901 nm, which was mainly responsible for the moisture content of vegetables. Hence, it is necessary to select the bands from the spectral range that could represent almost all of the FMs, as well as the vegetables. However, the models developed based on FT-NIR spectroscopy are only suitable for selecting the wavebands, and are not for real-time application. Hence, NIR imaging was used to verify the selected wavebands for identifying the FMs from fresh-cut vegetables in the following analysis.

### 3.6. Testing the Selected Wavebands Using NIR Imaging

**Figure 7:**
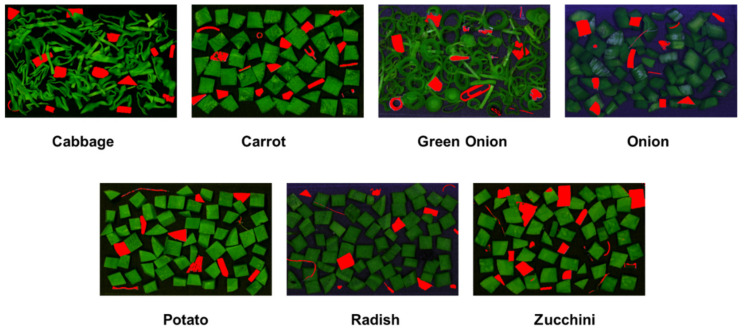
FMs detection images of fresh-cut vegetables using six wavebands.

Though the selected wavebands performed well in the NIR spectral data, the main challenge was to find out how the bands performed in multispectral imaging techniques. Therefore, the selected bands were tested using the NIR images of the vegetables and FMs (Figure 7). However, the wavebands selected using individual techniques were not enough to detect the FMs accurately by using the image. Hence, different combinations of the selected bands were explored to find out the best combination. Table 5 represents the number of correctly detected FMs in seven fresh-cut vegetables using three different combinations of bands from the selected wavelengths. The best accuracy was found using the combination of six bands (1150, 1400, 1450, 1731, 1880, and 1920), as shown in Table 5. These six wavebands represented almost all of the variables selected by the different wavelength selection techniques. Overall, 99 FMs were detected accurately among the 107 FMs in all of the vegetables. Table 5 also shows that the classification accuracy using five bands (1150, 1400, 1731, 1880, and 1920) was only 74.8%, whereas the accuracy became 92.5% after using six bands, and it did not improve after adding one more, i.e., seven bands (1150, 1400, 1450, 1731, 1880, 1920, and 2114).

However, the classification accuracy (92.5%) using the NIR imaging of selected bands was lower than the FT-NIR spectroscopy because the spectra were measured separately for the vegetables and FMs using FT-NIR spectroscopy, while for NIR imaging, the data were collected simultaneously by mixing the FMs with the vegetables. In addition, the spectral resolution of FT-NIR spectroscopy (0.6 nm at 1250 nm) was much higher than in the NIR imaging system (5.876 nm), and the instrumental set-up of FT-NIR spectroscopy was very compacted, which reduced the environmental effect and instrumental noise. In contrast, the noise produced from the instrument and the environment might affect the NIR images. Besides, the FMs, which had similar intensity with the background, were removed during the background removal process.

## 4. Conclusions

The results obtained from the PLS-DA models confirm that NIR spectroscopy has good potential for distinguishing FMs from fresh-cut vegetables with almost 100% accuracy. In this work, seven types of vegetables and various FMs were used to explore the waveband regions that would successfully separate all types of FMs from different varieties of vegetables. The effective wavebands were selected using WRC, VIP, SFS, SPA, and iPLS algorithms to identify the FMs accurately. The models developed using the selected wavebands, which were around 99% less than the total variables, on average, were almost similar to the original models. Therefore, the selected wavebands were used to test their effectiveness for real-time application using NIR imaging, and finally, six bands were selected after trying different combinations of bands. The test results showed an FMs detection accuracy of 92.5% for all seven fresh-cut vegetables. However, in the future, more research should be conducted to increase accuracy by increasing the spectral resolution of the NIR camera and by selecting a suitable background that can easily differentiate all kinds of FMs from vegetables. Overall, the results demonstrated good potential for designing an NIR multispectral imaging system to distinguish FMs from different types of fresh-cut vegetables.

## Figures and Tables

**Table 1:** List of potential foreign materials used in this study.

Name of FMs	Type	Source
Paper	Different color	Printing paper, books, dairy, packaging paper, packaging boxes, sticky notes, etc.
Plastics	ABS	Various processed food packages, bottles, industrial waste plastics, laboratory waste plastics, etc.
	LDPE	
	HDPE	
	PET	
	PS	
	PC	
	PP	
	Nylon	
Rubber	Different color	Nondestructive biosensing lab, Chungnam National University
Tissue	Different source	Nondestructive biosensing lab, Chungnam National University
Threads	Different color	Nondestructive biosensing lab, Chungnam National University
Stone	Different size and place	Daejeon, South Korea
Wood	Processed	Toothpick, earbuds
	Raw	Plant stem
Cigarettes	Different brand	Different person
Insects	Mosquito	Environment
	Bee	
	Fly	
	Ant	
	Spider	
	Grasshopper	
	Butterfly	
	Others	
Human nail	Various size	Different person
Metal	Nuts, bolts, wires, springs, foils, etc.	Nondestructive biosensing lab, Chungnam National University

**Table 2:** Summary of PLS-DA model on the identification of FMs in vegetables using different preprocessing methods.

		Total Number of Samples	Calibration (420 Samples)			Validation (180 Samples)			LVs
			Correctly Classified Fresh-Cuts	Correctly Classified FMs	Accuracy (%)	Correctly Classified Fresh-Cuts	Correctly Classified FMs	Accuracy (%)	
Normalization	Mean ^1^	600	196	224	100	84	96	100	4
	Max ^2^	600	196	224	100	84	96	100	5
	Range ^3^	600	196	224	100	84	96	100	6
	MSC ^4^	600	196	224	100	84	96	100	9
	SNV ^5^	600	196	224	100	84	96	100	6
Derivatives	SG1 ^6^	600	196	224	100	84	96	100	4
	SG2 ^7^	600	196	224	100	84	95	99.4	6
	Raw	600	196	224	100	84	96	100	5

^1^ Mean normalization; ^2^ maximum normalization; ^3^ range normalization; ^4^ multiplicative scatter correction; ^5^ standard normal variance; ^6^ Savitzky–Golay first derivative; ^7^ Savitzky–Golay second derivative.

**Table 3:** The selected wavelengths for identifying the FMs in fresh-cut vegetables using different variable selection methods.

Variable Selection Method	Selected Variable Number	Selected Wavelengths (nm)
WRC	5	1150, 1401, 1731, 1880, and 1920
VIP	10	1888–1901
SFS	3	1136, 1450, and 1921
SPA	4	1300, 1402, 1925, and 2114
iPLS	3	1094, 1343, and 1958

**Table 4:** The results of the test data set for identifying the FMs in fresh-cut vegetables using the new PLS-DA models with selected wavebands.

Model	Samples Used	No. of Correctly Detected Vegetables	Sensitivity (%)	No. of Correctly Detected FMs	Specificity (%)	Accuracy (%)
WRC-PLS-DA	300	200	100	100	100	100
VIP-PLS-DA	300	200	100	100	93	97.67
SFS-PLS-DA	300	200	100	100	100	100
SPA-PLS-DA	300	200	100	100	100	100
iPLS-DA	300	200	100	100	100	100

**Table 5:** FMs detection accuracy in fresh-cut vegetables using the selected variables in NIR imaging.

Vegetables	Total No. of FMs	5 Bands		6 Bands		7 Bands		False Positive (6 Bands)
		No. of Correctly Identified FMs	Total Accuracy (%)	No. of Correctly Identified FMs	Total Accuracy (%)	No. of Correctly Identified FMs	Total Accuracy (%)	
Cabbage	13	12	74.77	13	92.5	13	92.5	3
Carrot	18	15		17		17		
Green onion	13	9		13		13		
Onion	16	10		14		14		
Potato	12	7		11		11		
Radish	16	12		13		13		
Zucchini	19	15		18		18		
